# How sign language expertise can influence the effects of face masks on non-linguistic characteristics

**DOI:** 10.1186/s41235-022-00405-6

**Published:** 2022-06-23

**Authors:** Wee Kiat Lau, Jana Chalupny, Klaudia Grote, Anke Huckauf

**Affiliations:** 1 General Psychology, Institute of Psychology and Pedagogics, Ulm University, Albert-Einstein-Allee 47, 89081 Ulm, Germany; 2 Competence Centre for Sign Language and Gesture (SignGes), RWTH Aachen, Theaterplatz 14, 52062 Aachen, Germany; 3 Regionalstelle Bad Nauheim, Autismus-Therapieinstitut Langen, Karlstraße 57 – 59, 61231 Bad Nauheim, Germany

**Keywords:** Emotion and personality recognition, Facemasks, Deaf, Hearing, Sign language

## Abstract

Face masks occlude parts of the face which hinders social communication and emotion recognition. Since sign language users are known to process facial information not only perceptually but also linguistically, examining face processing in deaf signers may reveal how linguistic aspects add to perceptual information. In general, signers could be born deaf or acquire hearing loss later in life. For this study, we focused on signers who were born deaf. Specifically, we analyzed data from a sample of 59 signers who were born deaf and investigated the impacts of face masks on non-linguistic characteristics of the face. Signers rated still-image faces with and without face masks for the following characteristics: arousal and valence of three facial expressions (happy, neutral, sad), invariant characteristics (DV:sex, age), and trait-like characteristics (attractiveness, trustworthiness, approachability). Results indicated that, when compared to masked faces, signers rated no-masked faces with stronger valence intensity across all expressions. Masked faces also appeared older, albeit a tendency to look more approachable. This experiment was a repeat of a previous study conducted on hearing participants, and a post hoc comparison was performed to assess rating differences between signers and hearing people. From this comparison, signers exhibited a larger tendency to rate facial expressions more intensely than hearing people. This suggests that deaf people perceive more intense information from facial expressions and face masks are more inhibiting for deaf people than hearing people. We speculate that deaf people found face masks more approachable due to societal norms when interacting with people wearing masks. Other factors like age and face database’s legitimacy are discussed.

**Supplementary Information:**

The online version contains supplementary material available at 10.1186/s41235-022-00405-6.

## Significance statement

Perceiving facial expressions is impaired by face masks. Sign language users are known to process facial characteristics perceptually and linguistically. Investigating effects of face masks on evaluation of emotional expressions, of bodily characteristics, and of personality traits in a sample of 59 deaf signers who were born deaf, signers replicate typically reported effects of face masks. Ratings from signers were lower for masked faces than for no-masked ones. This reduction was stronger as compared to those from hearing people. Thus, face masks influence how hearing and deaf people perceive faces.

## Reading the face and effects of face masks

The face is important for social communication. Key features of the face like the mouth and nose regions facilitate social interactions by helping us interpret face movements (Seamon et al., 1978). Much of the information from the face is communicated perceptually. For instance, facial expressions convey an individual’s emotional state such as fear, anger, or sadness (Dadds et al., 2006; Wegrzyn et al., 2017) and are communicated rapidly when looking at the face (Batty & Taylor, 2003). Besides the perceived emotional state of another person, we sometimes perceive bodily characteristics such as sex or age when looking at the face (Brown & Perrett, 1993; Nkengne et al., 2008). These characteristics are referred to as invariant since they remain relatively stable over time. Individual facial features also suggest certain traits. For example, facial features can influence how attractive, trustworthy, or approachable the face appears (Perrett et al., 1999; Scheib et al., 1999; Todorov et al., 2008; Vernon et al., 2014). Therefore, we perceive various pieces of information about a person when looking at the face.

A face may be perceived differently when a face mask is present for hearing people. In some cases, happy and angry expressions become mistaken as neutral expressions, while disgusted expressions become confused with angry expressions (Carbon, 2020). Such mistakes at reading the facial expressions of another person can quickly become awkward or even dangerous. Also, it is more difficult to recognize the face’s sex when the face is occluded by masks as compared to the full face (Freud et al., 2020). Besides falsely identifying emotional expressions or sex, facial trustworthiness can also become misjudged due to face masks. Low-trusting faces appeared more trustworthy when the face wore a face mask (Marini et al., 2021). Misattributing trustworthiness to untrustworthy strangers may not be socially desirable, especially when the misattribution occurs simply due to the presence of face masks. There was a greater reduction to interpersonal distances (i.e., higher approachability) for strangers wearing face masks as masked faces appeared more trustworthy than full faces (Cartaud et al., 2020). As a final note, masked faces appeared healthier and more attractive, presumably because asymmetrical and/or unattractive regions of the lower face region were occluded by face masks, resulting in an increase in the perceived attractiveness of the face (Kamatani et al., 2021). In sum, studies suggest that face masks could modify the perceived characteristics of another hearing person.

A recent study investigated the relationship between face masks and various perceived characteristics derived from looking at the face (Lau & Huckauf, 2021). In that study, 196 hearing participants rated sex, age, attractiveness, trustworthiness, approachability, arousal, and valence between faces with and without face masks. Results showed that face masks reduced the perceived intensity of facial expressions. Moreover, faces with face masks appeared older and less attractive than fully visible faces. The findings suggested that face masks played a role in influencing various perceptual impressions from the face, at least in hearing people population.

### Face processing in deaf people who use sign language

Deaf people who use sign language, or signers, do not explore facial information in the same way than hearing non-signers (Watanabe et al., 2011). In signing, non-manual facial gestures from the mouth and brows convey not only emotional markers but also varying degrees of lexical, prosodic, and grammatical information (Brentari & Crossley, 2002; Liddell, 2003). For example, the mouth gesture of a smile can be semantically aligned to nouns, adjectives, and simple verbs (Bank et al., 2011; Johnston et al., 2016), but also include ancillary emotional cues which are processed by signers perceptually. Deaf signers match hearing non-signers in the recognition of facial expression of emotion (Rodger et al., 2021), although for them facial expressions in the first place provide grammatical and syntactic markers. Thus, some studies argue that such gesticulations may also include ancillary emotional cues which are processed by signers perceptually.

Deaf signers do not process emotional facial expressions the same way as hearing people. Facial expressions carry semantic, iconic, and compositional information for deaf people (see review by Elliot & Jacobs, 2013). Imaging studies have documented that deaf people recruit face- and language-processing regions when viewing an emotional face. In contrast, hearing people only used the face-processing regions (McCullough et al., 2005; McCullough & Emorey, 1997). Signers may also process emotional facial expressions differently than hearing people due to how the face is fixated. Signers look in equal amounts at the top and bottom halves of a face when judging the face’s identity and emotion. Hearing people spent more time looking at the top half of the face to judge identity and the bottom half of the face to judge emotions (Letourneau & Mitchell, 2011).

Some studies suggest that emotional recognition in deaf people is poorer than hearing people. For example, deaf people identified disgust better than hearing people (Corder, 1988), and hearing people identified happiness better than deaf people (Weisel, 1985). However, the differences in emotional recognition between deaf people and hearing people are related to categorical perception of facial expressions and language acquisition.

Categorical perception is a perceptual bias. It occurs because the perceptual sensitivity to a stimulus is strongest at the boundary between two categories, than within any single category (Etcoff & Magee, 1992). Hearing people are highly biased when judging facial expressions. Subtle differences in facial expressions within an emotion category are almost impossible to discriminate due to this bias. Conversely, deaf people only show this bias when processing the linguistical component of the facial expression. There is no bias when deaf people evaluate the affective component of the face (McCullough & Emmorey, 2009).

Emotional recognition for deaf people is poor due to language acquisition. For instance, deaf children performed poorer that recognizing facial expressions than hearing children because of delayed linguistic acquisition. Importantly, poorer recognition was only found for fear, surprise, and disgust (Sidera et al., 2017). These expressions were considered difficult to recognize in children. Nevertheless, signing enhances the accuracy to recognize a face, with some speed trade-off (Stoll et al., 2018).

The iconicity of certain gestures may simultaneously convey linguistic and perceptual information. The iconic aspects of a sign affect the strength of semantic relations within a sign concept. Semantically related concepts which are reinforced by the iconic aspects of a specific gesture have a very close or strong connection to the related referent. When specific characteristics and attributes of a referent are highlighted in an iconic sign or its non-manual companions, the related concepts become reactivated, and the semantic relation is reinforced. For example, in German Sign Language, the “curved beak” is representative of the sign “eagle.” Thus, the beak gets a special relevance for the semantic concept “adler” because it is highlighted. Empirical studies show that the beak is semantically more strongly connected to the concept “eagle” than e.g., the “wing.” Thus, the iconicity of manual and non-manual signs which are derived perceptually facilitate semantic processing (Grote & Linz, 2003; Vinson et al., 2015).

There is evidence that facial information for sign language is processed perceptually and semantically in a blended manner. Grote and Linz (2003) demonstrated that the relation between semantic structure and iconic aspects of linguistic form can be interpreted as an effect of language modality on semantic conceptualization. This signified that the lexical and perceptual processes of mouth movements are integrally constitutive of sign language use. Undoubtedly, sign language is a conglomeration of complex processes blending perceptual and linguistic information (Bank et al., 2016). Despite the influence of iconicity, no studies have examined how mouth movements, integrated in the semantic concept of certain referents, affect the processing of faces covered by face masks.

### Face masks between deaf signers and hearing people

Deaf signers and hearing people can look at a face to extract various visual cues to facilitate social communication. Face masks could disturb such visual cues and lead to communication breakdown. One key difference is that hearing people can always fall back on auditory information as secondary cues for further clarifications. This often pits people who are deaf in situations where an ability to distinguish minute changes in the face becomes crucial for maintaining effective communication. Small changes in facial configurations is also a fundamental prerequisite for functioning sign language (Stokoe Jr, 2005). Face regions undisturbed by face masks, such as the eyes and eye region, may still be processed with more detail in deaf people than in hearing people. For example, the eyebrow region conveys syntactic information in sign language (De Vos et al., 2009; Weast, 2011). This suggests that the effects of face masks on face processing may differ between the deaf and the hearing.

Deaf signers may not be as susceptible as people who hear, to the perceptual disturbances from face masks besides linguistic impairments. Signers have higher precision than non-signers at differentiating bodily gestures visually (Poizner, 1983) due to a greater development of specific cognitive competences and structures which enhance visual-spatial abilities. The enhanced ability is the result of greater demands during the production and comprehension of grammatical constructions in sign language. Consequently, signers often excel in various processes like as creating and rotating mental images (Emmorey et al., 1993), reacting to visual events, processing, and attending to visual information (Neville & Lawson, 1987a, 1987b, 1987c; Pavani & Bottari, 2012), and processing haptic spatial information (van Dijk et al., 2013).

The enhanced visual-spatial abilities of signers suggests that signers may be more resilient against face mask effects when perceiving characteristics of a face. This would be true for information largely undisturbed by face masks. For example, signers should be equally adept at identifying the sex and age of a person since such characteristics are perceived by looking at the brows or wrinkles (Brown & Perrett, 1993; Nkengne et al., 2008). This would also apply for expressions with universal meaning which can be interpreted without reference to culture and language (Wierzbicka, 1999). Signers may be more adept than hearing people at detecting such universal information, or at identifying subtle differences in the face. Signers should experience greater disturbances at evaluating face characteristics when important cues are occluded. As previously discussed, the mouth region communicates important semantic and perceptual information. Since face masks occlude the mouth, signers should experience larger impairments at processing facial characteristics derived from the mouth for masked faces. Hence, depending on which face region a characteristic is perceived, one might suspect that signers possess the capacity to accurately evaluate a face regardless of face masks, or experience greater disturbances due to face masks. Such data would also be informative in understanding how the combined perceptual and linguistic components of signing contribute to face processing.

### Aims and goals

The following experiment assessed how face masks influenced signers’ ability to evaluate faces by repeating a previous work (Lau & Huckauf, 2021). The previous work involved 196 hearing participants rating a set of eight black and white faces presented in random order. Half the faces wore a face mask, and the other half were full faces (no-masked). Masked faces always appeared masked in the experiment, and no-masked faces remained unmasked throughout the experiment. Participants rated emotional expressions of arousal and valence (happy, neutral, sad faces), invariant characteristics of sex and age (neutral faces), and trait-like characteristics of attractiveness, trustworthiness, and approachability (neutral faces).

The current study involved 102 deaf participants who repeated the same experiment. Not all deaf participants reported use of sign language. Some deaf participants also reported prior hearing and/or speaking experiences before losing hearing. It is known that prior hearing experiences play an important role in sign language. Children with hearing loss in late childhood were more proficient in sign language than children born deaf (Mayberry, 1993). Moreover, hearing abilities facilitate sign language acquisition (Morgan et al., 2007). This alludes to the possibility that prior hearing and/or speaking experiences could influence the study results. Thus, we reported data from 59 deaf signers who were born deaf to rule out hearing and speaking experiences as potential confounds. Signers assessed a series of faces with and without face masks. The following characteristics were rated for randomly presented faces: Signers evaluated facial expression intensities for valence and arousal, as a direct replication of the previous study (Lau & Huckauf, 2021). Valence and arousal scales were adapted from the Self-Assessment Manikin (SAM) scale (Bradley & Lang, 1994). The rationale of using the scale was to reveal small effects of face masks on emotion recognition.

We expected signers to experience greater reductions in the perceived intensity of arousal and valence when rating faces with face masks because prominent visual and semantic cues of the mouth were occluded by face masks. We did not expect signers to experience differences between faces with and without face masks when rating other characteristics due to their sensitivity in face reading. We then investigated effects of face masks on face evaluation between signers from this study and hearing people from a previous study. Effects of face masks were quantified as the difference in ratings between no-masked faces and masked faces.

## Method

### Participants

A total of 102 deaf participants took part in this study. Participants were recruited in accordance with the declaration of Helsinki and the ethical rules of the German Research Foundation (DFG). Fifty-nine reported to be born deaf, while 47 reported losing hearing abilities later in life. We reported data from participants who were born deaf so that the influence of hearing or speaking experiences could not be a factor in the study. The following data belonged to these 59 participants. The gender distribution leaned slightly in favor of females (*N* = 36, 61%) than in males (*N* = 22, 37.30%). One participant identified as a diverse individual (1.7%). Most participants (*N* = 23, 39%) were between 30–39 years of age, followed by < 30 years (*N* = 19, 32.20%), then 50–59 years of age (*N* = 9, 15.25%), and finally 40–49 years of age (*N* = 8, 13.56%). Most participants reported using sign language daily (*N* = 57, 96.61%). The remaining participants did not self-report sign language use since the question was not compulsory. Of the participants who reported sign language use, the majority native language was German sign language (*N* = 47, 79.66%). Data from deaf participants (*N* = 2, 3.39%) who did not report sign language used was not discarded as this would decrease statistical power.

The following summarizes the data concerning hearing participants from Lau and Huckauf (2021). 196 participants (90 females, 99 males, three diverse) completed the study. 124 (63.26%) participants reported < 30 years old, 35 (17.87%) < 40 years old, 21 (10.71%) < 50 years old, and 16 (8.16%) > 50 years old.

### Stimuli

**Fig. 1:**
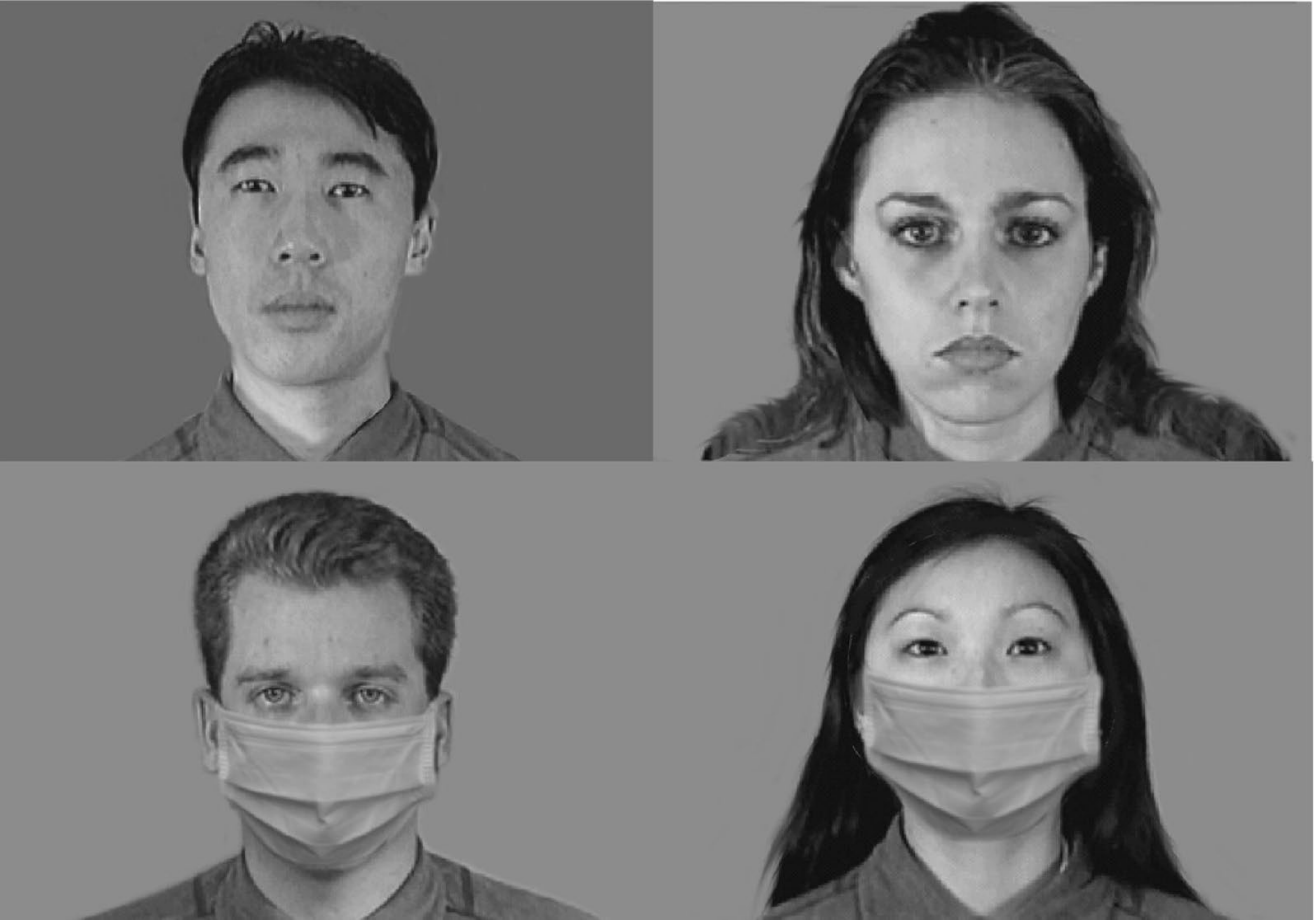
Sample neutral faces used in the study. There were eight faces in the experiment. Four of which were shown here. All faces were photoshopped with face masks. Signers saw masked and no-masked faces belonging to different facial models. Signers never saw the same face both masked and no-masked during the study

The study was a direct replication of previous work using Latin-square design (Lau & Huckauf, 2021). All stimuli used in this study were taken directly from the previous work and used the same way. Briefly, eight grayscale facial models (four females, four males; each gender comprising two Asian and two Caucasian faces) were taken from the *Montreal Set of facial displays of emotion* (MSFDE) facial database (Beaupré et al., 2000). Faces depicted happy, neutral, or sad expressions. Face masks were added onto the faces via Photoshop (Fig. 1). Signers saw faces with face masks and faces without face masks. For the remaining manuscript, we refer to the faces as no-masked faces and masked faces.

Due to the COVID-19 pandemic, access to a laboratory was heavily regulated. To ensure that the study provided the greatest accessibility to deaf people, we conducted an online study via the online survey platform, EFS Survey by Questback GmbH (2021).

### Design

**Fig. 2:**
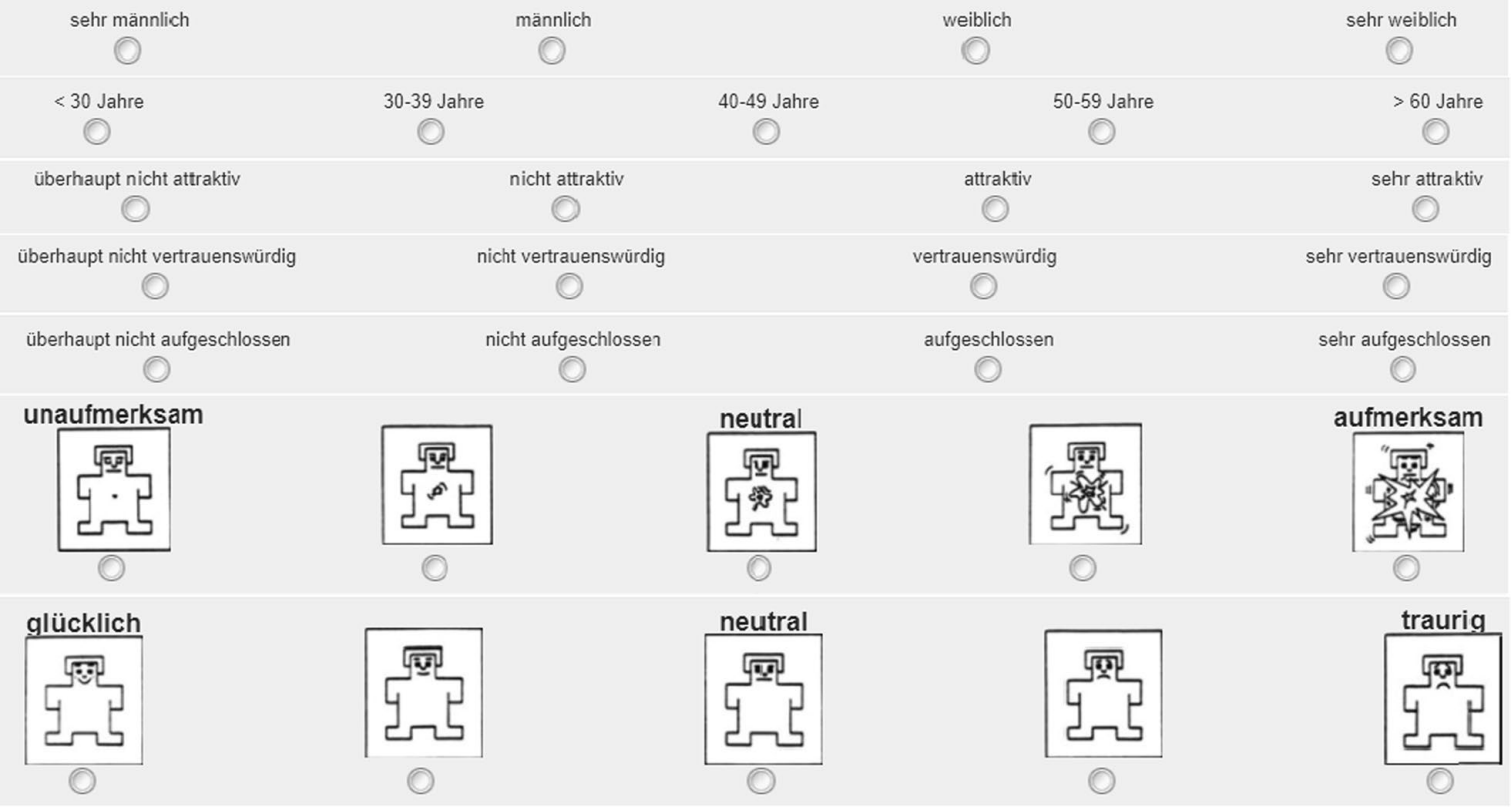
Scales used in the study. The characteristics measured were (top to bottom): sex, age, attractiveness, trustworthiness, approachability, arousal, and valence. The scales were written in German and corresponded to the English version used in a previous study (see Lau & Huckauf, 2021)

The study design was the same as the previous work. There were two blocks. Block 1 measured invariant characteristics (sex, age) and trait-like characteristics (attractiveness, trustworthiness, approachability) of neutral faces. Except for age, the ratings in block 1 were given on a 4-point Likert scale (Fig. 2). The lowest value on the scale, i.e., 1-point, corresponded to the least agreement to a characteristic (e.g., not attractive at all) or the youngest rating (< 30 years old). The highest value on the scale corresponded to the strongest agreement (e.g., very attractive) or the oldest ratings (> 60 years old). The scale for sex ranged from 1 = surely male to 4 = surely female. Sex ratings were recorded during analysis such that higher values represented better accuracy. Therefore, in block 1, participants rated how female or male the faces appeared, the age, and how attractive, trustworthy, and approachable the neutral faces appeared.

Block 2 measured emotional expressions of valence and arousal of happy, neutral, and sad faces. The ratings in block 2 were given on a 5-point Likert scale. The ratings for arousal ranged 1 = calm, 3 = neutral, 5 = excited. The ratings for valence ranged 1 = happy, 3 = neutral, 5 = sad. The scales used in the previous study were in written English. However, in this study, the scales were translated into written German. Therefore, in block 2, participants rated the perceived valence and arousal of happy, neutral, and sad faces.

In Block 1, the neutral faces were from four models (two females, two males) ✕ two Mask (no-masked, masked faces) = eight faces. Faces for each characteristic were presented randomly. Signers evaluated all eight faces before proceeding to rate a different characteristic. The characteristics in Block 1 were presented in the following order: sex, age, attractiveness, trustworthiness, and approachability. The total trials for block 1 were eight faces ✕ five characteristics = 40 trials. In Block 2, signers saw happy, neutral, and sad faces. There were four models (two females, two males) ✕ two Mask (no-masked, masked faces) ✕ three expressions (happy, neutral, sad) = 24 faces. Faces were presented randomly as Latin-square design. Signers evaluated arousal for all 24 faces before rating valence. Therefore, the total trials for block 2 were 24 faces ✕ two emotional expressions (arousal, valence) = 48 trials.

There were two parallel versions of the study, Version A and Version B. Masked faces in one version were no-masked ones in the other version. Masked faces remained masked throughout the study. Similarly, no-masked faces remained no-masked throughout the study. Signers only completed one version. Consequently, signers never saw the same face with and without face masks.

There was an instructional video at the beginning of the study in German sign language (GSL). The video is found in Additional file 1: appendix S1.mp4. The purpose of the video was to explain to the participants about the experiment. Participants were told in GSL about data confidentiality, informed consent, that they could interrupt and end the experiment without consequences, and that there were no right or wrong answers. The video ended by asking participants to share the study link with their friends and families, before thanking them for their participation. The intelligibility of the video was validated by some (*N* = 3) deaf volunteers before data collection began.

### Procedure

The procedure was the same as in Lau and Huckauf (2021). The study was conducted online from November 6, 2020, to November 30, 2020. Signers were recruited through social media networks in the form of written German and a study link. Upon clicking on the study link, participants saw the introduction page with some German text. The text provided participants information concerning data confidentiality and informed consent. Participants provided informed consent by clicking on the “I agree” button and then clicking on the *next* button to begin the study. Signers were presented immediately with a short instructional video (Additional file 1: appendix S1.mp4) in German sign language about the experiment. The video explained about informed consent. Specifically, participants were instructed that the data will be collected and analyzed anonymously, that they could interrupt the experiment any time without any consequences, and that there were no right or wrong responses. The video ended by asking participants to share the study link to their friends and families before thanking them for participation. Participants were randomly assigned to one of two versions of the study once the video ended. All other instructions in the experiment existed as written German, exactly as in the previous study.

A practice trial appeared after the video ended. The purpose of the practice trial was to familiarize signers with the assessment scales and the tasks. During the practice trial, signers rated the attractiveness of a female face by selecting one of the points on the 4-point Likert scale. This face never appeared again for the remaining study. Responses from the practice trial were discarded from the analysis. The practice trial ended once a response was given.

Block 1 began immediately after the practice trial ended. An instruction screen was presented. Signers always saw this instruction screen which explained the next characteristic they had to rate. The scale associated with the characteristic was also shown in the instruction screen. Signers must acknowledge that they understood the task before proceeding. Faces were then presented randomly one after another. For each trial, a face appeared with the following prompt “Dieser Person erscheint mir …” (This person seems to me …) positioned below the image. The scale was located below the prompt. There was only one face on screen which remained until a rating was given. A different face was then shown, and signers repeated the evaluation process until each face was rated once per characteristics. There was no time limit to rate the face. The characteristics were presented in the following order: sex, age, attractiveness, trustworthiness, approachability.

Block 2 started immediately after block 1. Again, an instruction screen was always presented explaining the task and which characteristics to rate. Signers acknowledged that they understood the task before continuing with the study. For each trial, like in block 1, a face appeared with the prompt “Dieser Person erscheint mir …” below the image. The scale to provide responses was located beneath the prompt. There was only one face which remained on screen until a response was given. The next face appeared immediately, and the process was repeated until all block 2 faces received one rating per characteristics. The characteristics were rated in the following order: arousal, valence. At the end of block 2, signers were presented demographic questions (sex, age, educational status, extent of hearing loss, native language, frequency of sign language use). Sex, age, and educational status were mandatory questions following the previous study. The other questions were additional ones to the replication. They were not mandatory as they could be perceived as sensitive topics. The average completion time of the whole survey was 16 min.

### Analysis

All analyses were conducted with SPSS in version 23 (IBM Corp. Released, 2015). The independent variables (IVs) were face masks (masked, no-masked), face’s race (Caucasian, Asian), and face’s gender (female, male). The dependent variables (DVs) were emotional expressions (valence and arousal) for happy, neutral, and sad faces, invariant characteristics (perceived sex, perceived age), and trait-like characteristics (perceived attractiveness, trustworthiness, and approachability). To avoid confusion between the IV face’s gender, and the DV sex, these variables were named: IV:gender and DV:sex.

The goal of the study was to investigate if face masks influence how signers rated various characteristics of another person. Preliminary descriptive statistics did not reveal strong biases toward any face stimuli. No single face stood out across all ratings, that is, no faces were rated higher than the other due to racial appearance (Caucasian or Asian faces) or due to IV:gender appearance (Female or male faces). This suggested that signers did not display gender or race biases when evaluating the face models. Thus, the face’s race and IV:gender were excluded in subsequent analyses.

Regarding emotional expressions, we evaluated the influence of face masks on the DVs arousal and valence. We ran two 2 × 3 repeated measures ANOVA for the factors mask (masked, no-masked), and facial expression (happy, neutral, sad), one for valence, one for arousal. The interaction effect between masks and emotional expressions were also analyzed. Post hoc comparisons were conducted for significant main effects using paired *t* tests with Bonferroni-corrected *ɑ* = 0.05/3 = 0.0167.

To investigate effects of face masks on ratings of invariant bodily characteristics (DV:sex, age), and of trait-like characteristics (attractiveness, trustworthiness, approachability), we ran paired *t* tests between masked and no-masked ratings for these five characteristics. The significance level for the paired *t* tests were conservatively corrected for using Bonferroni-corrected *ɑ* = 0.05/5 = 0.01. Therefore, *p* values ≤ 0.01 were regarded as significant.

An additional post hoc analysis was conducted to evaluate whether signers or hearing people experienced a larger influence from face masks when rating characteristics about the face. Data from hearing people was taken from a previous study (Lau & Huckauf, 2021). Since the current study was a repeat of the previous one, the variables collected in between the two studies were identical. All variables in both studies were the same: valence, arousal, DV:sex, age, attractiveness, trustworthiness, and approachability. The demographic variables were also identical for: gender and age. The analysis conducted in this study was the same as those in the previous study.

We first evaluated whether the demographics (i.e., gender and age) across the two samples differed from each other. Sample differences due to demographics would complicate how the results were interpreted, since any findings could simply be attributed to group differences. This evaluation was conducted using independent samples *t* tests on the gender and age variables. Levene’s test for equality of variances was calculated to test if the two samples were homogenous. The *t* tests were recomputed when homogeneity assumption was violated.

The effects of face masks were quantified by the rating differences between no-masked and masked faces. We subtracted no-masked face ratings with masked face ratings in the respective samples. Positive values indicated biases for no-masked faces and negative values reflected biases for masked faces.

We anticipated a potential confusion when interpreting face mask effects on valence ratings in happy faces. This was because low valence ratings reflected more positive valence. Assuming that an observer was perfect at rating valence, negative values from subtracting no-masked valence ratings with masked valence ratings would suggest a bias for no-masked faces instead of a bias for masked faces as defined previously. To avoid this confusion, we reversed coded valence ratings in happy faces before running the post hoc analysis. This aligned the interpretation of face mask effects on valence ratings: positive values reflect a bias for no-masked faces, and negative values reflect a bias for masked faces.

Face mask effects between hearing people population and signers were evaluated using independent samples *t* tests. Levene’s test for equality of variances was first computed to check if the comparisons between the two samples were homogenous. The *t* tests which did not assume homogeneity were recalculated whenever comparisons violated the homogeneity assumption.

## Results

### Emotional expression

**Fig. 3:**
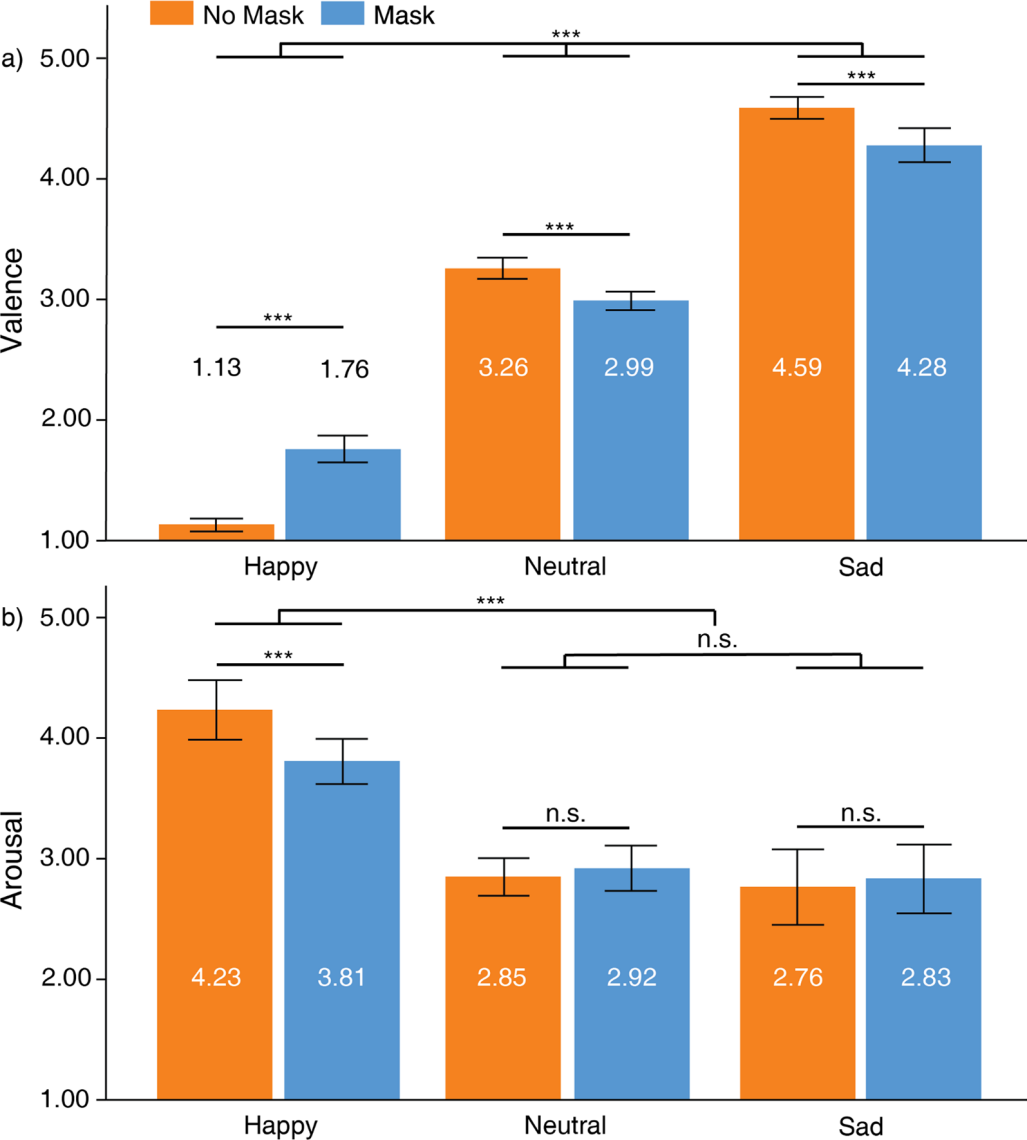
Ratings for **a** valence and **b** arousal. Higher values in **a** indicate more negative valence. Higher values in **b** indicate more arousal. Error bars depict standard error of means (SEM). ***p < .001, **p < .01, *p < .05

Valence ratings differed between the three facial expressions, *F*(2, 57) = 914.89, *p* < 0.001, partial *η*^2^ = 0.97 (Fig. 3a). Post hoc comparisons indicated that happy faces (*M* = 1.45, *SD* = 0.25) were rated happier than neutral faces (*M* = 3.13, *SD* = 0.27), *t*(58) =  − 36.32, *SD* = 0.36, *p* < 0.001 (Bonferroni-corrected *ɑ* = 0.0167), and sad faces (*M* = 4.44, *SD* = 0.38), *t*(58) =  − 42.26, *SD* = 0.54, *p* < 0.001 (Bonferroni-corrected *ɑ* = 0.0167). Neutral faces were also rated happier than sad faces, *t*(58) =  − 26.39, *SD* = 0.38, *p* < 0.001 (Bonferroni-corrected ɑ = 0.0167). There was no main effect of face masks on valence ratings, *F*(1, 58) = 0.25, *p* > 0.05, partial *η*^2^ = 0.004. The interaction between face masks and facial expressions was significant, *F*(2, 57) = 85.35, *p* < 0.001, partial *η*^2^ = 0.75. Post hoc comparisons revealed that no-masked happy faces (*M* = 1.13, *SD* = 0.20) were rated happier than masked happy faces (*M* = 1.76, *SD* = 0.43), *t*(58) =  − 10.99, *SD* = 0.44, *p* < 0.001 (Bonferroni-corrected *ɑ* = 0.0167). No-masked neutral faces (*M* = 3.26, *SD* = 0.34) were rated less happy than masked neutral faces (*M* = 2.99, *SD* = 0.29), *t*(58) = 6.21, *SD* = 0.34, *p* < 0.001 (Bonferroni-corrected ɑ = 0.0167). No-masked sad faces (*M* = 4.59, *SD* = 0.35) were rated less happy than masked sad faces (*M* = 4.28, *SD* = 0.54), *t*(58) = 4.71, *SD* = 0.51, *p* < 0.001 (Bonferroni-corrected *ɑ* = 0.0167). That is, emotional expressions of masked faces were rated as less intense than those of unmasked faces.

Arousal ratings differed between the three facial expressions, *F*(2, 57) = 62.38, *p* < 0.001, partial *η*^2^ = 0.69 (Fig. 3b). Post hoc comparisons indicated that happy faces (*M* = 4.02, *SD* = 0.77) were rated higher arousal than neutral faces (*M* = 2.88, *SD* = 0.54), *t*(58) = 11.25, *SD* = 0.78, *p* < 0.001 (Bonferroni-corrected *ɑ* = 0.0167), and sad faces (*M* = 2.80, *SD* = 1.08), *t*(58) = 6.14, *SD* = 1.53, *p* < 0.001 (Bonferroni-corrected *ɑ* = 0.0167). Arousal ratings did not differ between neutral faces and sad faces, *t*(58) = 0.54, *SD* = 1.24, *p* > 0.05. There was no main effect of face masks on arousal ratings, *F*(1, 58) = 2.72, *p* > 0.05, partial *η*^2^ = 0.05. However, the interaction between face masks and facial expressions was significant, *F*(2, 57) = 8.81, *p* < 0.001, partial *η*^2^ = 0.24. Post hoc comparisons revealed that no-masked happy faces (*M* = 4.23, *SD* = 0.95) were rated more aroused than masked happy faces (*M* = 3.81, *SD* = 0.72), *t*(58) = 4.69, *SD* = 0.70, *p* < 0.001 (Bonferroni-corrected *ɑ* = 0.0167). However, arousal ratings did not differ between no-masked neutral faces (*M* = 2.85, *SD* = 0.60) and masked neutral faces (*M* = 2.92, *SD* = 0.72), *t*(58) =  − 0.71, *SD* = 0.78, *p* > 0.05. There was also no differences in arousal ratings between no-masked sad faces (*M* = 2.76, *SD* = 1.20) and no-masked sad faces (*M* = 2.83, *SD* = 1.10), *t*(58) =  − 0.66, *SD* = 0.79, *p* > 0.05.

The results suggest an absence of strong face mask effects on valence and arousal. Perceived valence intensity was reduced in masked faces as compared to no-masked ones. However, signers remained adept at correctly identifying the facial expressions. Although happy faces appeared less aroused when masked, face masks did not influence the perceived arousal ratings for neutral and sad faces.

### Invariant bodily characteristics

**Fig. 4:**
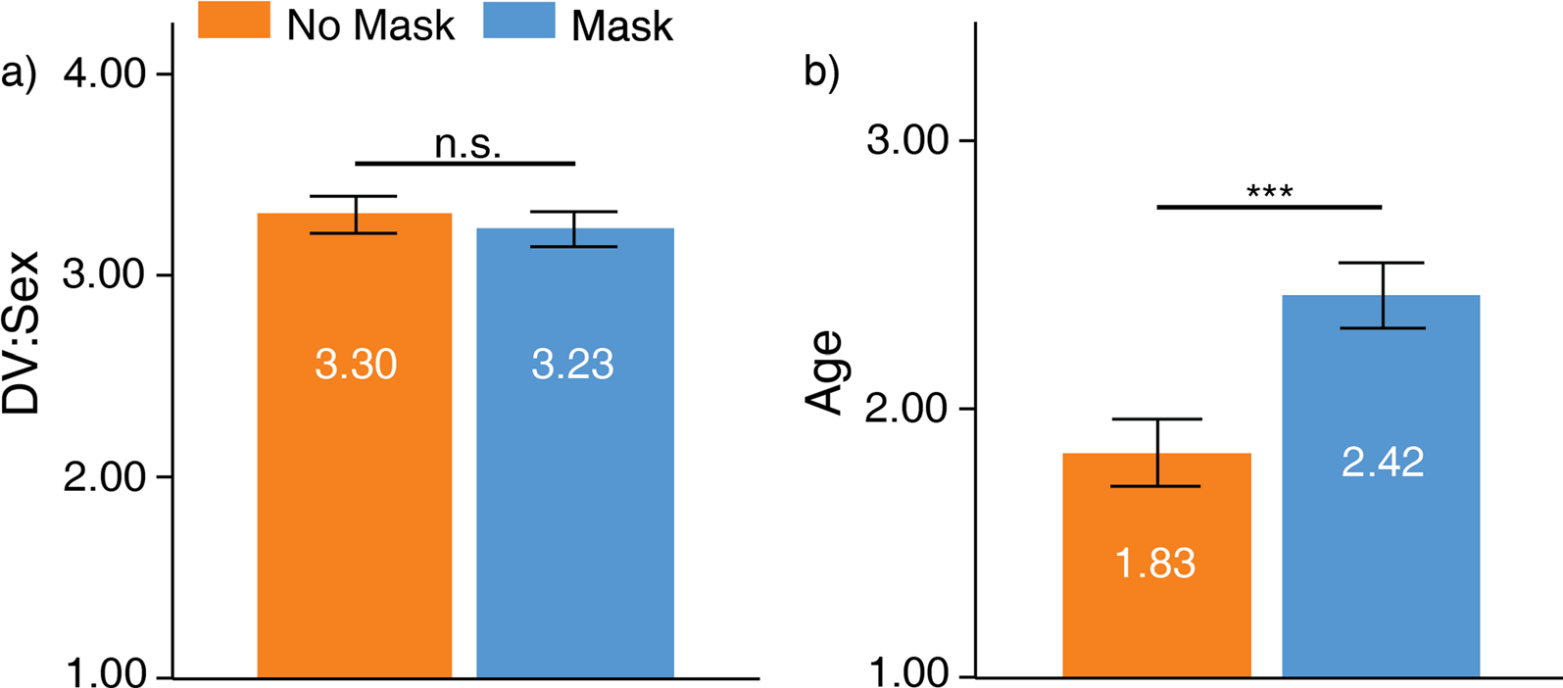
Ratings for **a** DV:sex and **b** age. Higher values in **a** indicate greater accuracy, while higher values in b) indicate older. Error bars indicate standard error of means (SEM). ***p < .001, **p < .01, *p < .05

Face masks did not influence DV:sex ratings, *t*(58) = 1.86, *SD* = 0.30, *p* > 0.05, Fig. 4a. Signers were accurate at rating both the DV:sex of no-masked (*M* = 3.30, *SD* = 0.35) and masked (*M* = 3.23, *SD* = 0.33) faces. Face masks influenced age ratings, *t*(58) =  − 7.66, *SD* = 0.59, *p* < 0.001 (Bonferroni-corrected *ɑ* = 0.01), Fig. 4b. Signers perceived no-masked faces (*M* = 1.83, *SD* = 0.48) much younger than masked faces (*M* = 2.42, *SD* = 0.47). Hence, face masks only influenced the perceived age of the face.

### Trait-like characteristics

**Fig. 5:**
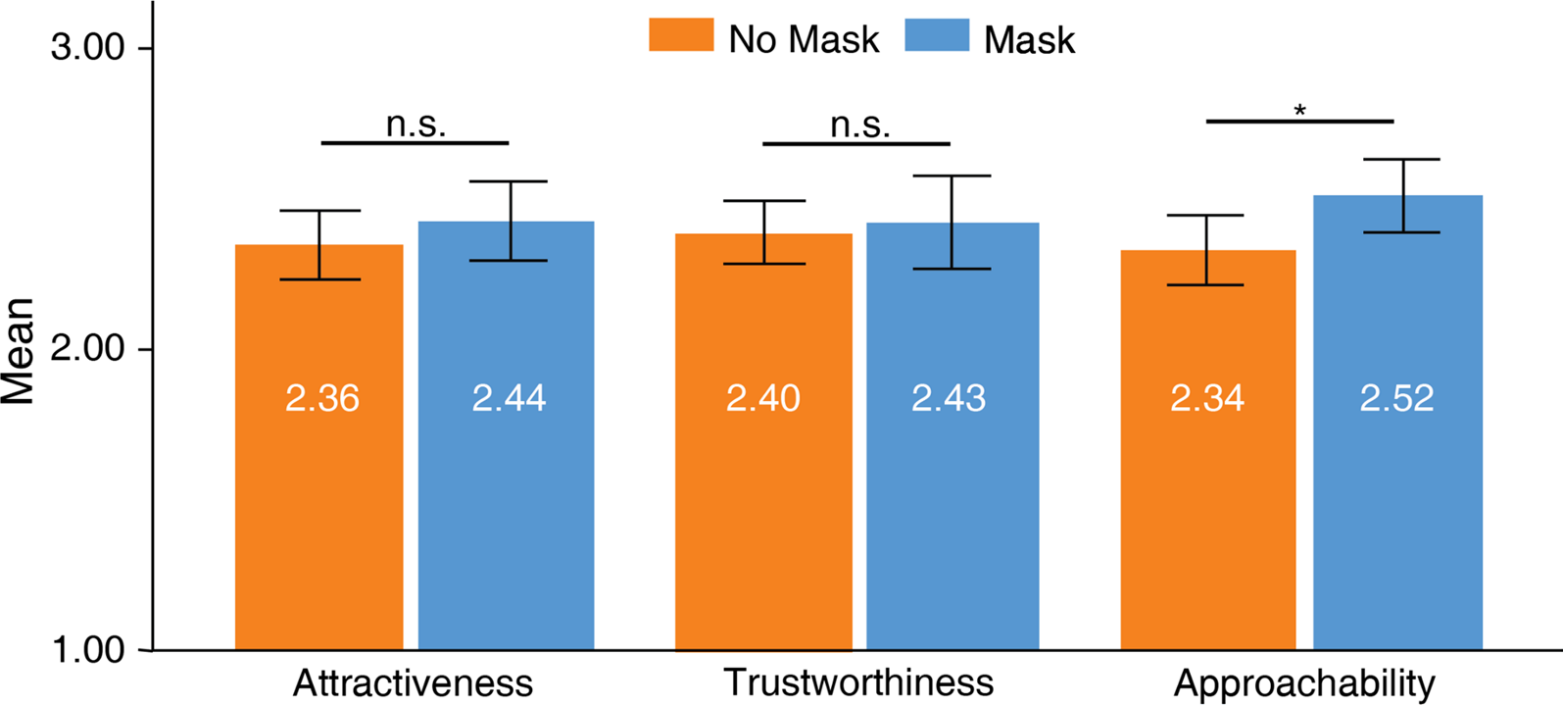
Ratings for trait-like characteristics. Higher values indicate greater agreements to the trait. Error bars indicate standard error of means (SEM). Approachability was considered nonsignificant (*p* = .013) because it did not survive Bonferroni correction, ɑ = .01. ***p < .001, **p < .01, *p < .05

Face masks did not influence attractiveness ratings (Fig. 5), *t*(58) =  − 1.18, *SD* = 0.53, *p* > 0.05. Signers did not perceive attractiveness differently between no-masked faces (*M* = 2.36, *SD* = 0.44) and masked faces (*M* = 2.44, *SD* = 0.51). Face masks did not influence trustworthiness ratings, *t*(58) =  − 0.42, *SD* = 0.62, *p* > 0.05. Signers perceived trustworthiness equally for no-masked faces (*M* = 2.40, *SD* = 0.41) and masked faces (*M* = 2.43, *SD* = 0.60). Face masks did not influence approachability ratings, *t*(58) =  − 2.58, *SD* = 0.54, *p* = 0.013 (Bonferroni-corrected *ɑ* = 0.01), since the comparison did not survive Bonferroni correction. This indicated that signers were indifferent to the approachability of no-masked (*M* = 2.34, *SD* = 0.45) and masked faces (*M* = 2.52, *SD* = 0.47).

In summary, signers’ perception of attractiveness, trustworthiness, and approachability was not affected by face masks.

### Signers versus hearing: demographics

Homogeneity assumption for participants’ gender was not violated *F*(1, 248) = 2.85, *p* > 0.05. There were no significant differences in gender between the samples, *t*(248) = 1.86, S.E. = 0.08, *p* > 0.05, 95% C.I. = [-0.01 0.30]. Homogeneity assumption for participants’ age was not violated, *F*(1, 248) = 0.04, *p* > 0.05. Age differed significantly between the samples, *t*(248) = 3.38, S.E. = 0.15, *p* < 0.001, 95% C.I. = [0.21 0.79]. Specifically, the hearing sample had more younger participants (*N* = 124, 63.26%) < 30 years old than the deaf sample (*N* = 19, 32.20%).

### Signers versus hearing: emotional expressions

**Fig. 6:**
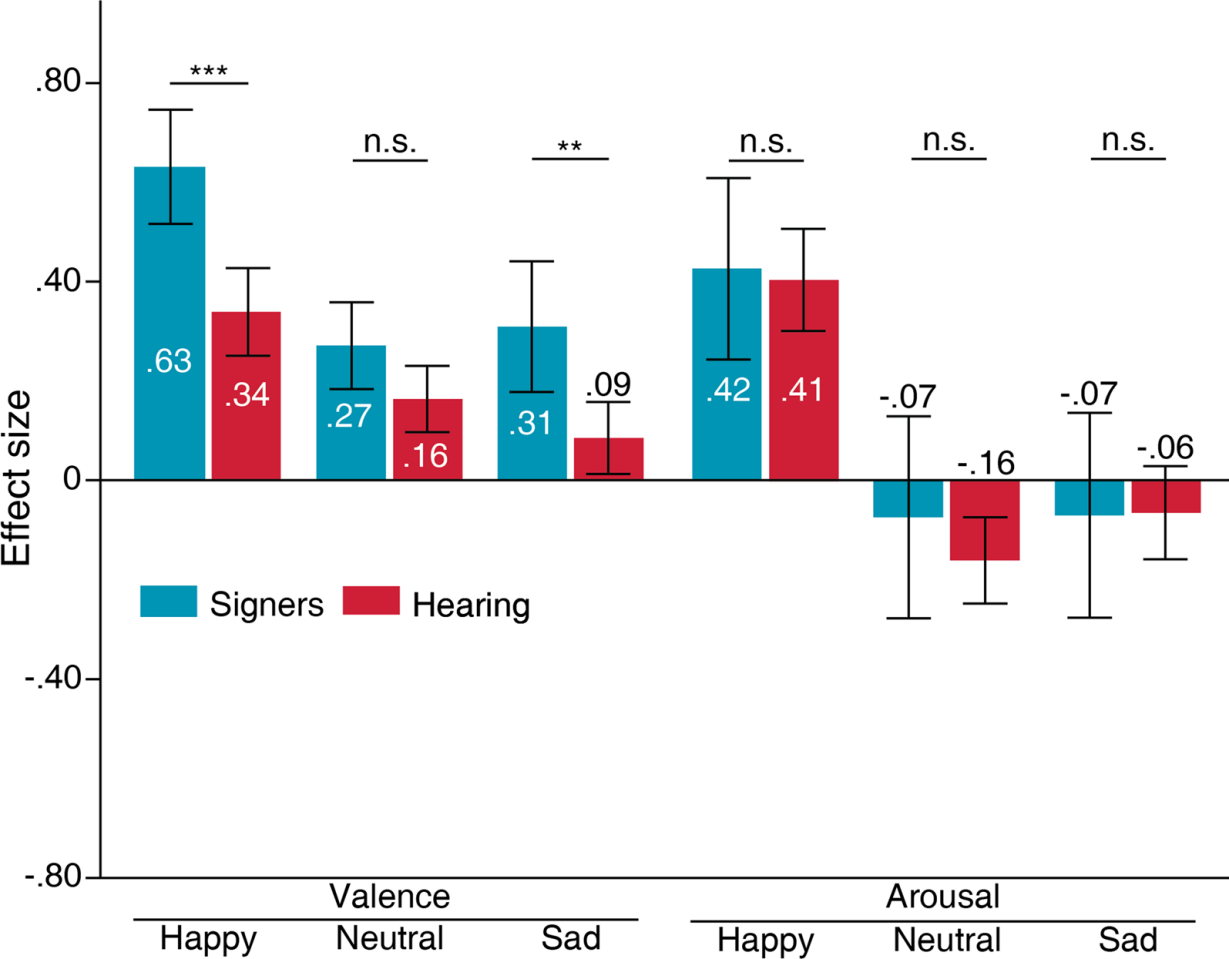
Effects of face masks between signers and hearing people for valence and arousal ratings. Positive values indicate greater bias for no-masked faces. Negative values indicate greater bias for masked faces. Error bars indicate standard error of means (SEM). ***p < .001, **p < .01, *p < .05

The effects of face masks on valence ratings for happy expressions violated homogeneity assumptions, *F*(1, 248) = 5.37, *p* < 0.05 (Fig. 6). The following comparison assumed non-homogenous variance between signers and hearing people. According to the analysis, signers (*M* = 0.63, *SD* = 0.44) were more biased by full faces than hearing people (*M* = 0.34, *SD* = 0.62) when rating valence in happy faces, *t*(134.67) =  − 4.01, S.E. = 0.07, *p* < 0.001, 95% C.I. = [0.15 0.45]. The effects of face masks on valence ratings for neutral expressions violated homogeneity assumptions, F(1, 248) = 7.48, *p* < 0.01. The following comparison assumed non-homogenous variance between signers and hearing people. From the comparison, neither signers (*M* = 0.27, *SD* = 0.34) nor hearing people (*M* = 0.16, *SD* = 0.47) were grossly biased by face masks when rating valence in neutral faces, *t*(133.53) = 1.95, S.E. = 0.06, *p* > 0.05, 95% C.I. = [-0.002 0.22]. The effects of face masks on valence ratings in sad faces did not violate homogeneity assumptions, F(1, 248) = 0.17, *p* > 0.05. Signers (*M* = 0.31, *SD* = 0.51) were more biased by the full face than hearing people (*M* = 0.09, *SD* = 0.51) when rating valence in sad faces, *t*(248) = 2.96, S.E. = 0.08, *p* < 0.01, 95% C.I. = [0.07 0.37].

The effects of face masks on arousal ratings for happy expressions did not violate homogeneity assumptions, F(1, 248) < 0.001, *p* > 0.05. Neither signers (*M* = 0.43, *SD* = 0.70) nor hearing people (*M* = 0.41, *SD* = 0.72) were greatly influenced by face mask effects when rating arousal of happy faces, *t*(248) = 0.21, S.E. = 0.11, *p* > 0.05, 95% C.I. = [-0.19 0.23]. The effects of face masks on arousal ratings for neutral expressions violated homogeneity assumptions, F(1, 248) = 4.51, *p* < 0.05. The following comparison assumed non-homogenous variance between signers and hearing people. From the comparison, neither signers (*M* =  − 0.07, *SD* = 0.78) nor hearing people (*M* =  − 0.16, *SD* = 0.61) were grossly biased by face mask effects when rating arousal in neutral faces, *t*(81.06) = 0.78, S.E. = 0.11, *p* > 0.05, 95% C.I. = [-0.13 0.31]. The effects of face masks on arousal ratings in sad faces did not violate homogeneity assumptions, F(1, 248) = 1.83, *p* > 0.05. Neither signers (*M* =  − 0.07, *SD* = 0.79) nor hearing people (*M* =  − 0.06, *SD* = 0.65) were greatly biased by face masks when rating arousal in sad faces, *t*(248) =  − 0.05, S.E. =  − 0.10, *p* > 0.05, 95% C.I. = [-0.21 0.20].

In summary, the bias experienced by signers when evaluating valence is the same as hearing people. Importantly, signers showed stronger biases for the full face than hearing people. Both signers and hearing people did not experience strong face mask biases when rating arousal.

### Signers versus hearing: invariant bodily characteristics and trait-like characteristics

**Fig. 7:**
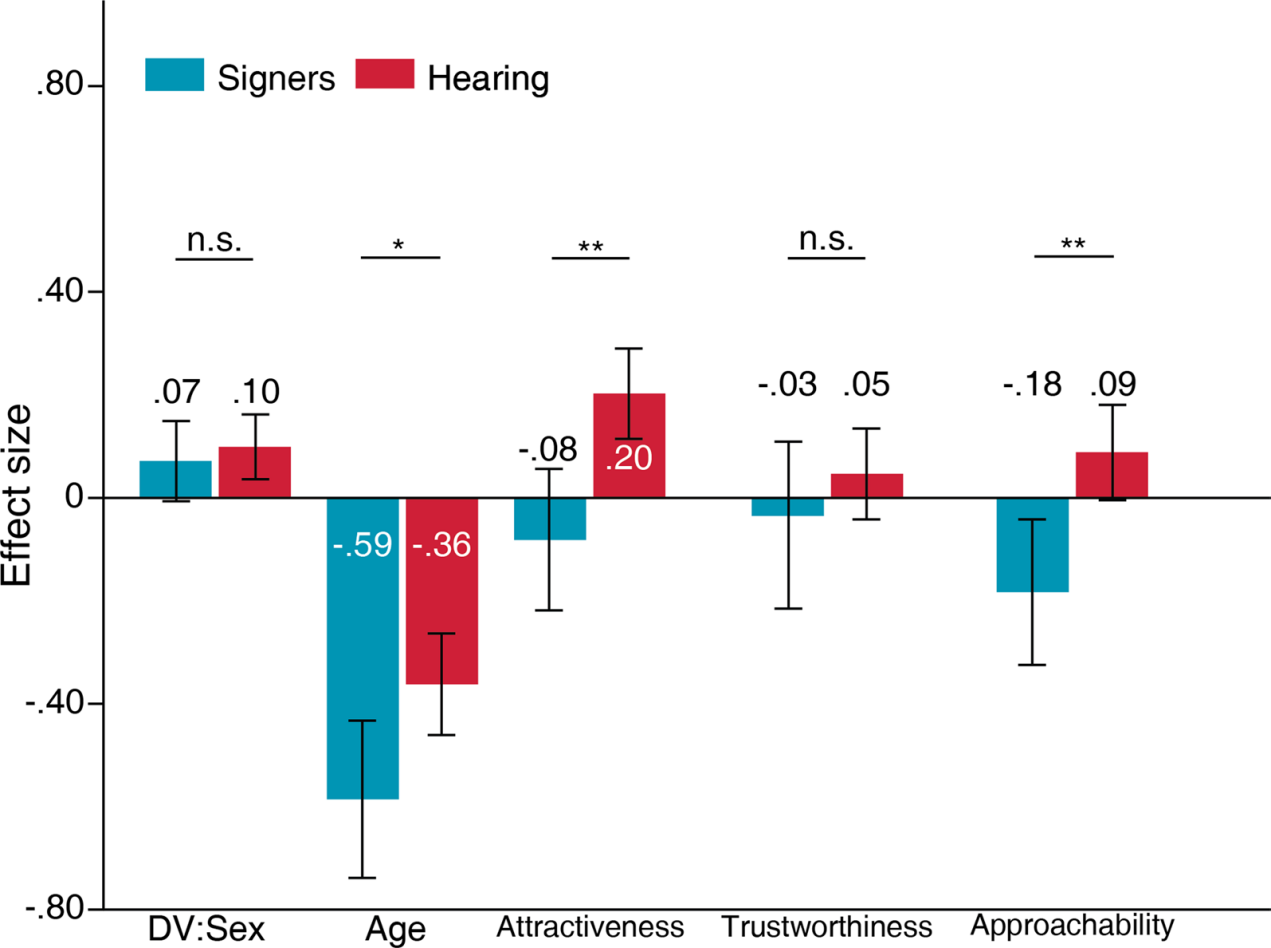
Effects of face masks between signers and hearing people for DV:sex, age, attractiveness, trustworthiness, and approachability ratings. Positive values indicate greater bias for no-masked faces. Negative values indicate greater bias for masked faces. Error bars indicate standard error of means (SEM). ***p < .001, **p < .01, *p < .05

The effects of face masks on DV:sex ratings violated homogeneity assumptions, F(1, 248) = 4.71, *p* < 0.05. The following comparison assumed non-homogenous variance between signers and hearing people (Fig. 7). According to the comparison, neither signers (*M* = 0.07, *SD* = 0.30) nor hearing people (*M* = 0.10, *SD* = 0.44) were subjected to greater influence when rating the face’s sex, *t*(143.68) =  − 0.55, S.E. = 0.05, *p* > 0.05, 95% C.I. = [-0.13 0.07]. The effects of face masks on age ratings also violated homogeneity assumptions, F(1, 248) = 4.23, *p* < 0.05. The following comparison assumed non-homogenous variance between the two samples. The analysis indicated that signers (*M* =  − 0.58, *SD* = 0.59) were more biased by masked faces than hearing people (*M* =  − 0.36, *SD* = 0.69) when rating age, *t*(112.32) =  − 2.45, S.E. =  − 0.09, *p* < 0.05, 95% C.I. = [-0.40 − 0.04].

The effects of face masks on attractiveness ratings did not violate homogeneity assumptions, F(1, 248) = 1.15, *p* > 0.05. Signers (*M* =  − 0.08, *SD* = 0.53) were more biased by face masks than hearing people (*M* = 0.20, *SD* = 0.62) when rating attractiveness of faces, *t*(248) =  − 3.19, S.E. =  − 0.09, *p* < 0.01, 95% C.I. = [-0.46 − 0.11]. The effects of face masks on trustworthiness ratings did not violate homogeneity assumptions, F(1, 248) = 1.14, *p* > 0.05 (Fig. 7). Neither signers (*M* =  − 0.03, *SD* = 0.62) nor hearing people (*M* = 0.05, *SD* = 0.62) experienced greater influence from the face masks when rating trustworthiness, *t*(248) =  − 0.88, S.E. =  − 0.09, *p* > 0.05, 95% C.I. = [-0.26 0.10]. The effects of face masks on approachability ratings did not violate homogeneity assumptions, F(1, 248) = 0.92, *p* > 0.05. Signers (*M* =  − 0.18, *SD* = 0.54) were more biased by face masks than hearing people (*M* = 0.09, *SD* = 0.65) when rating approachability, *t*(248) =  − 2.92, S.E. =  − 0.09, *p* < 0.01, 95% C.I. = [-0.45 − 0.09].

In short, signers experienced a greater bias from face masks when rating age, attractiveness, and approachability as compared to hearing people. In contrast, hearing people experienced greater biases from full faces when rating attractiveness and approachability.

## Discussion

The face conveys information about a person which we rely on in everyday life. In this study, we examined if various facial characteristic ratings differed between masked and no-masked faces in signers. Differences between masked and no-masked faces occurred mainly in perceived valence and in perceived age of the stimuli. Both effects also differed significantly from those observed in hearing people.

### Impacts of blended processing of perceptual and linguistic information

The face is used in sign language to convey both perceptual and linguistic information. Facial features, like eyebrows and wrinkles, may tell us perceptually about a person’s sex, age, or personality traits (Nkengne et al., 2008; Todorov et al., 2008). Signers then utilize the eyebrow region to gain syntactic information for sign language use (De Vos et al., 2009; Weast, 2011). Facial expressions may articulate perceptually a person’s current emotional state (Dadds et al., 2006; Wegrzyn et al., 2017), but the linguistic component from mouthing and mouth gestures fail when face masks occlude the mouth (Balvet & Sallandre, 2014; Johnston et al., 2016).

Face masks hinder communication in people who are deaf by partially blocking perceptual and linguistic information from the face. Our results corroborated with this argument. We first demonstrated that signers also experienced a reduction in valence intensity ratings for masked faces when compared to no-masked faces. This also replicated data from hearing people. It could be argued that the replication was evidence showing that face masks interfered with emotion recognition when evaluating a face perceptually. The evidence showing that face masks hindered the linguistic component of the face came from the rating differences between signers and hearing people. Specifically, signers attributed higher ratings for no-masked faces than hearing people, especially for happy and sad faces. This indicated that signers perceived more intense information from facial expressions than hearing people. This could be due to the additional linguistic processing which signers use to decode perceptual and linguistic information from a face. Consequently, signers experienced greater face evaluation disturbances due to face masks.

Our results may indicate findings specific only to German signers. Just like any spoken language, sign languages across the world are not universal. German signers may place stronger emphasis on lipreading because Germany has a strong oral education for deaf students in school (oralism). This means that the conclusions derived from this study could be specific for GSL, and/or other sign languages with stronger focus on oral education. Nevertheless, we would like to stress that it is not important to link lipreading specifically to GSL signers since all deaf people and hearing-impaired people rely on lipreading and mimics to some extent in their communication. Thus, the broader conclusion is that face masks affect communication in all deaf and hearing-impaired people.

### The influence of daily habits and face masks in deaf people

Since the start of the pandemic, deaf people experienced struggles which hearing people are unaware of. One struggle was to understand what was happening when there was a sudden surge in mask wearing. Signers did not understand that a pandemic had occurred because there were no proper hand signs or gestures to describe the virus (Garg et al., 2021). Public information concerning the pandemic or guidelines to protect oneself from the virus was also not immediately available to deaf and hearing-impaired people (Panko et al., 2021; Park, 2020).

Communication was a struggle for deaf people and those who are hearing-impaired. This was due to the face mask requirements and social distancing. The use of face masks was prevalent to curb the spread of air-borne viruses, such as the COVID virus (Tabatabaeizadeh, 2021; WHO, 2020). Social distancing was recommended to avoid close contact with those potentially infected. Lipreading was no longer possible when face masks cover the mouth. Communication was further complicated when clinicians and clients must now distance themselves (Chodosh et al., 2020). Some of the struggles experienced by deaf and hearing-impaired individuals could be remedied with better instructions, signages, and technology (McKee et al., 2020). However, this discussion is beyond the scope of the manuscript.

The use of face masks is an Achilles’ heel for all deaf and hearing-impaired people. It is undeniable that face masks render lipreading impossible. This is probably the main reason why deaf people consider face masks unpleasant as communication is dramatically reduced (Grote & Izagaren, 2020; Sheik-Ali et al., 2021). Since the pleasantness of a face (i.e., how positive the face appears) results in greater approachability and attraction (Neta et al., 2021), one may expect masked faces to be perceived as less approachable since facial features that make a face appear pleasant are occluded by face masks. However, our data revealed that signers born deaf displayed a tendency to rate masked faces as more approachable. It is contradicting to find this result in deaf people, since face masks are unpleasant as they hinder communication from face- and lipreading. However, there is an ambiguity in how approachability was measured. We did not dissociate between facial pleasantness and the pleasantness of interacting with a face wearing face masks. This was because the current study was identical to a previous study conducted on hearing participants (Lau & Huckauf, 2021), where approachability was assumed to be facial pleasantness of a face with face mask. Thus, we can only speculate why deaf people found masked faces more approachable using the same assumption (i.e., facial appearance pleasantness).

Evidence in increased approachability for hearing people occur due to a gradual acceptance and transformation based on rules enforced by the society and from social interactions. Hearing people were more willing to reduce their interpersonal distances to strangers with face masks than without face masks (Cartaud et al., 2020). When an individual adheres to instructions enforced by authorities, this could manifest into actual daily habits. This is also a reflection of our (i.e., the authors) experiences as hearing individuals. As more people behave the same way, such behaviors become defined as social norms. Social norms promote the individual to adopt the group’s behavior, such as wearing face masks (Barceló & Sheen, 2020; Carbon, 2021). Although people who are deaf may find face masks unpleasant, the norm of interacting with others wearing face masks may change opinions surrounding face masks, and face masks would appear more pleasant. However, we cautiously draw this speculation as how hearing people would behave in relation to social norms.

### The legitimateness of face databases

Face databases hardly provide any ground truth on facial expressions. Some face databases provide standardized ratings to facilitate post hoc comparisons between sampled data and the database data. However, we cannot be certain on how authentic and intense an emotion was expressed on a given photo. The database used in the current study did not provide any information about the exact age of the models. It was unclear whether the higher valence ratings demonstrated by signers could be interpreted as better face recognition performance in comparison to hearing people. One might speculate that databases show exaggerated versions of facial expressions like acting in a theater piece. This would suggest that in addition to the perceptual information, the exaggeration amplified the linguistic information about facial expressions. Incidentally, the exaggerated expressions facilitated signers on their identification of various emotions. However, the exaggeration could not overcome the interference of face masks such that masked faces would be perceived along the same facial expression magnitude as no-masked versions.

### Age and face masks

We found significant age differences between the two samples. There were more younger participants in the hearing sample than in the deaf sample. This implied that our findings could have been partly due to age-related biases. Age biases occur when the task requires age estimation. Younger participants tend to overestimate the age of old faces as compared to older participants (Voelkle et al., 2012). This could explain why hearing people overestimated the age of no-masked faces as compared to deaf people (see Table 1). However, this would not explain why deaf people showed a larger bias when rating the age of masked faces. A possible explanation could be that age perception changes depending on the context and what was covering the face (Thorley et al., 2022).

**Table 1:** Ratings between signers and hearing people

Characteristics	Signers				Hearing			
	No-mask		Mask		No-mask		Mask	
	*M*	*SD*	*M*	*SD*	*M*	*SD*	*M*	*SD*
Emotional expressions								
Valence								
Happy	1.13	.20	1.76	.43	1.49	.85	1.82	.71
Neutral	3.26	.34	2.99	.29	3.15	.39	2.99	.44
Sad	4.59	.35	4.28	.54	4.22	.75	4.14	.74
Arousal								
Happy	4.23	.95	3.81	.72	4.20	1.06	3.80	.90
Neutral	2.85	.60	2.92	.72	2.60	.66	2.76	.58
Sad	2.76	1.20	2.83	1.10	2.69	1.13	2.75	1.17
Invariant								
DV:Sex	3.30	.35	3.23	.33	3.76	.43	3.66	.46
Age	1.83	.48	2.42	.47	1.99	.59	2.36	.63
Trait-like								
Attractiveness	2.36	.44	2.44	.51	2.65	.48	2.45	.47
Trustworthiness	2.40	.41	2.43	.60	2.64	.47	2.60	.46
Approachability	2.34	.45	2.52	.47	2.70	.50	2.61	.46

Data from hearing people was adapted from Lau and Huckauf (2021)

It is undeniable that face masks interfere with face perception and emotional recognition across ages. School children experienced difficulties in recognizing faces. This occurred for both upright and inverted faces with face masks (Stajduhar et al., 2022). Interestingly, one study suggested that children may not be as susceptible to emotional recognition of masked faces. This was because children were equally accurate at distinguishing emotions of partially occluded faces, independent of the device which was occluded the face (Ruba & Pollak, 2020). In contrast, adults could experience confusion when recognizing emotions of masked faces (Carbon, 2020), or experience a reduction in the perceived intensity of the emotions (Lau & Huckauf, 2021). Hence, a person’s age could influence how the face and the emotional expression is perceived.

### Limitations and future directions

There were some limiting factors regarding the generalizability of our data. Personal attitudes toward the coronavirus and the mask obligation were not measured. In fact, there were various mixed public opinions toward mask obligations especially during the early phases of the pandemic. According to the literature, personal attitudes influence behavior, even toward wearing face masks and other face coverings (Boer & Fischer, 2013; Fischer et al., 2012). The increased frequency of watching masked faces, and other social norms, could have conditioned signers on their responses. Therefore, it would be advantageous to replicate the experiment again later to investigate whether the current findings remain stable over time.

The study was conducted using still images of facial expressions. Real-world social interaction requires the observer to process faces dynamically. These include viewing faces at varying angles and different expressions. Replicating the current results using dynamic faces is important as some authors argue that people who are deaf exhibit larger effects than hearing people (Krejtz et al., 2020). Dynamic videos can offer more valid assessments of facial expressions than still pictures due to the naturalness of the expressions (Sato & Yoshikawa, 2004). For example, Duchenne smiles have specific facial musculature contractions (Ekman et al., 1990). These smiles are iconic in expressing happiness for still images. However, dynamic Duchenne smiles may appear ingenuine depending on the smile duration (Krumhuber & Kappas, 2005). This could influence the intensity of the perceived happiness expression.

## Conclusion

Face masks impair emotional communication processes in both signers who were born deaf and in hearing people. Signers showed a reduction in valence ratings for masked faces in contrast to no-masked faces. The effect was strongest for happy and sad expressions (i.e., the most expressive faces). When compared to hearing people, signers experienced greater disturbances due to face mask effects. This supports the argument that deaf people extract more intense information from facial expressions.

## Supplementary Information

**Additional file 1.** Instructional video in German Sign Language (GSL) regarding the experiment.

## Data Availability

The datasets generated and/or analyzed during the current study are available in the Open Access Repositorium der Universität Ulm und Technischen Hochschule Ulm (OPARU), https://oparu.uni-ulm.de/xmlui/handle/123456789/38966
